# Properties of bone from Catfish heads and frames

**DOI:** 10.1002/fsn3.974

**Published:** 2019-03-02

**Authors:** Peter J. Bechtel, Michael A. Watson, Jeanne M. Lea, Karen L. Bett‐Garber, John M. Bland

**Affiliations:** ^1^ Food Processing and Sensory Quality Research Unit, Agricultural Research Center, Southern Regional Research Center USDA New Orleans Louisiana

**Keywords:** analysis, bone composition, catfish by‐products, fish bone

## Abstract

The objective of this paper was to evaluate methods of producing purified Catfish bone fractions from Catfish frames and heads and determine the composition of the purified bone fraction. Fresh samples of Catfish frames and heads were obtained from a large commercial Catfish processor. Triplicate samples were processed for all treatments. Two methods were developed to remove nonbone tissue from the frames: (a) use of a proteolytic enzyme to digest the nonbone tissues and (b) after boiling the frames, removal of the nonbone tissues with high‐pressure water. The ash, protein, and lipid contents of unprocessed dried frames were 17%, 33%, and 41%, respectively. After the enzymatic or high‐pressure water treatment processes, the frame bone compositions for the two processes were 62% and 54% ash, 35% and 33% protein, and 9% and 2% lipid, respectively. Bone from both processing treatments had a calcium content of 21%–25%, phosphorus content of 10%–11%, and contents of magnesium, manganese, zinc, and nickel were increased. Hydroxyproline content increased from 4% of the amino acids in the untreated bone to 7%–8% for the processed treatments. Tissues were removed from Catfish heads by digestion with a proteolytic enzyme and collection of the bone with a sieve. After the longest digestion period, dried head bone was 51% ash, 38% protein, and 7% lipid. The amino acid profiles showed high levels of hydroxyproline and lower levels of many essential amino acids. With increased enzymatic hydrolysis time, percent calcium and phosphorus increased. Results from this study will be used in the development of new value‐added food and feed ingredients from Catfish bone.

## 1 INTRODUCTION

There were 301 million pounds of Catfish produced in 2014 (Hanson & Sites, 2012). Whole, dressed Catfish is further processed into common usable forms, which include regular fillets, shank fillets, fillet strips, and nuggets (Silva & Dean, 2001). In addition, Catfish by‐product account for 55%–65% of the whole fish mass. Processing of Catfish results in the production of a large amount of fish waste. Depending on what product is being produced, the waste (or by‐product) can account for >60% of the harvested weight of the fish and consist of varying amounts of heads, viscera, frames, skin, and lesser amounts of blood and fins (Crapo & Bechtel, 2003; Yin et al., 2010). Currently, Catfish by‐product from larger processing operations is combined and sold to rendering plants where by‐products are used to produce protein meals and oils for use as feed ingredients. Some smaller processors chose to dispose of the waste, make fertilizers, or utilize directly as a feed ingredient. Few Catfish processors further process or manufacture other products from their Catfish waste, causing most of the material to be shipped to rendering plants. Little use has been made of individual parts such as skin, or viscera components such as stomachs and livers.

Progress has been made in evaluating Catfish by‐product components such as using fish skin for gelatin production (Jiang, Shaoyang, Du, & Wang, 2010; Yang et al., 2007; Yin et al., 2010), Vietnamese Catfish meals (Nguyen, Lindberg, & Ogle, 2007), Catfish oil (Sathivel, Prinyawiwatkul, Grimm, King, & Lloyd, 2002), Catfish oil extraction (Sathivel, Yin, Prinyawiwatkul, & King, 2009), Catfish protein and hydrolysates (Davenport & Kristinsson, 2011; Theodore, Raghavan, & Kristinsson, 2008; Yin, Wan, Pu, Bechtel, & Sathivel, 2011), mince from frames (Hoke, Jahncke, Silva, Hernsberber, & Suriyaphan, 2000), minced belly flap meat (Wiles, Green, & Bryant, 2004), and Catfish roe (Sathivel, Yin, Bechtel, & King, 2009). Bechtel et al. (2017) provided comparisons of the major Catfish by‐products that are commercially available, for both channel and hybrid Catfish, including frames and heads (without treatment).

Frames from Channel Catfish account for 18% of the total fish weight (Woodruff, 1984) and are produced from the headed and gutted fish as the fillets are cut from the backbone. Frames consist of the backbones and rib bones and attached skeletal muscle, adipose, and connective and nerve tissues remaining after the removal of the fillets from the headed and gutted fish. Typically, Catfish frames are used to make Catfish meal and oil that are used as animal feed ingredients (Lovell, 1973). However, an excellent use for Catfish frames would be the development of value‐added products. Specifically, bone meal derived from Catfish frames could be used to develop calcium‐rich mineral supplements for humans or animals. Catfish have large heads which are approximately 20%–25% of the weight of the fish (Bosworth, Wolters, Silva, Chamul, & Park, 2004) and are a good source of bone.

Using Catfish frames and heads to develop a calcium‐rich supplement has the potential to combat osteoporosis and improve bone health in the elderly. Various studies indicate that the consumption of calcium has the advantage of enhancing the structural integrity of bone, and consumption of calcium has the advantage of enhancing the structural integrity of bone. There are a number of studies on the chemical and physical properties of fish bone. Toppe, Albrektsen, Hope, and Aksnes (2007) evaluated the chemical properties of bones from 8 different species of fish. Bones were from the frames and heads, and were removed after boiling of the tissue. This study reported differences in the lipid content of bone from different species and amino acid analysis, and element content was also reported. Other studies on properties of fish bone include those by Istiqlaal (2017) and Techochatchawal, Therdthai, and Khotavivattana (2009). Characterization and preparation of ultrafine and nano fish bone from silver carp have been reported by Wu, Zhang, Wanga, Mothibe, and Chen (2012); Yin, Du, Xhang, and Xiong (2016) and Yin, Park, and Xiong (2015). Venkatesan et al. (2015) reported chemical and physical properties of nanohydroxyapitite derived using alkaline hydrolysis process from salmon fish bone. Ren et al. (2012) reported using used 5 different proteases to hydrolyze channel Catfish bones to generate antibacterial agents. Chemical properties of Pacific cod and salmon bone from frames were reported by Bechtel (2003).

To increase the value of by‐product, it is imperative to first know the details of the physical and chemical composition of the raw material. The objective of this paper was to evaluate methods of producing a purified Catfish bone fractions from Catfish frames and heads and determine the composition of the purified bone fractions.

## 2 MATERIALS AND METHODS

### 2.1 Catfish frames

Catfish frames were acquired from a commercial Catfish processing facility in Mississippi and held in a −20°C freezer untilled thawed at 4°C. Frames also had some fins and ribs attached. Viscera consisted of the stomach, intestine, internal fat pad, some liver and testes, and egg when present. For the control treatment, 740 g of Catfish frame was weighed and dried in a Cyclone convection oven (model GDCO‐E, Baker's Pride Oven Company, Cheyenne, WY) for 24 hr at 66°C. After drying, Catfish frames were ground using a Waring Pro meat grinder (model WPG200SA, Waring Commercial, Torrington, CT) fitted with a plate having 0.25‐inch‐diameter holes. Catfish frames were then freeze milled using a Spex Sample Prep freezer mill (model 6870D, Spex Sample Prep, Metuchen, NJ) and stored in a −70°C freezer until needed. All treatments were replicated three times.

The pressure wash treatment used 740 g of Catfish frames which were placed in boiling water in a Groen Steam Kettle (Model TDA/1‐40) for 20 min. Catfish frames were then removed from the Groen Kettle and placed on a drying rack and pressure washed (Ryobi, model RY14122, Techtronic Industries, Anderson, SC) for 2 min per side. After pressure washing these frames, they were oven dried for 24 hr at 66°C, freeze milled, and stored at −20°C.

Catfish frame enzyme treatments consisted of 740 g of Catfish frames grounded with a Hobart grinder with a plate having 0.25‐inch holes. After grinding, approximately 740 g of deionized water was added to the ground frames and heated to 50°C for 15 min. After equilibration, 355 µl of Alcalase (Protease from *Bacillus lichenformis*, 2.4 U/g, Sigma Chemical, St. Louis) was added and the mixture stirred with an overhead propeller for 50°C for 30 or 60 min. The enzyme was inactivated by heating to 95°C for 20 min. The mixture was then permitted to cool down to room temperature, and the bone was separated from the hydrolysate by filtering through a 35 mesh stainless steel wired screen. It was washed three times with DI water, dried in a Cyclone convection oven at 66°C, and freeze milled.

### 2.2 Catfish heads

Catfish heads were acquired from a commercial Catfish processing facility in Mississippi. Heads were held in a −20°C freezer until thawed at 4°C. Catfish heads contained the heart and some of the liver. Heads were ground (model 7548, The Biro Mfg. Co., Marblehead, OH) first through a plate with 1‐inch and then 0.5‐inch‐diameter holes. Ground Catfish head material was separated into 300 g portions, placed in Ziploc bags and held in a −70°C freezer (So Low Environmental, Cincinnati, OH). All enzyme treatments were performed in triplicate using 300 g of semithawed Catfish head that was mixed with 300 g of deionized water and homogenized in a Waring Blender (model 38BL61) for 2 min. For all treatments, the Catfish head homogenate was preheated to 50°C using a microwave (model NN‐SN936B, Panasonic Appliances, Shanghai, CH). After attaining the 50°C temperature, 355 µl of Alcalase (2.4 U/g) was added to the Catfish head homogenate and placed in a shaker (Labline Instruments Inc., Melrose Park, IL) set at 110 rpm for 0, 5, and 30 min at 50°C. At the appropriate time, the hydrolyzed samples were put into a microwave and rapidly brought to 95°C and then placed on a hot plate to maintain a temperature of 95°C for 20 min to inactivate Alcalase.

All nonenzyme‐treated samples were subjected to the same procedures except no Alcalase enzyme was added to the homogenates. After the 95°C treatment, homogenates were filtered through a 35 mesh stainless steel sieve screen to separate the bone fragment material from hydrolysate liquids. The separated bone fragments were washed with 200 g of deionized water, and the bone fragments were dried in a Cyclone electric convection oven for 24 hr at 66°C. After drying, bone samples were weighed and freeze milled. All samples were stored in a −70°C freezer until further analyzed.

### 2.3 Proximate analysis

Moisture and ash contents were determined using AOAC methods #952.08 and #938.08, respectively (AOAC, 1990). Nitrogen content was accessed by pyrolysis with a LECO FP‐2000 nitrogen analyzer (LECO Co., St. Joseph, MO). Protein content was calculated as 6.25 times % N. Total lipid content was determined gravimetrically by the method of Dodds, McCoy, Geldenhuys, Rea, and Kennish (2004) after extraction with an Accelerated Solvent Extractor (ASE; ASE 250, Dionex, Sunnyvale, CA) using methylene chloride. Solvent was removed under a N_2_ gas stream at 40°C using a TurboVap LV (Caliper Life Sciences) in preweighed vials. The remaining traces of solvent were removed under vacuum until constant weight was achieved.

Conversion of wet weight proximate percentages to a dry weight percentage was accomplished in Excel. A new set of proximate numbers equal to the wet weight values was created, and a third set of proximate numbers was created equal to the second set multiplied by the ratio of the sum of the original wet weight proximate values to the sum of the second set. Then, the Excel solver function (data tab) was used to change the moisture value of the second set so that the moisture value of the third set equaled the value needed (solver parameters used an objective set so the moisture value of the third set of values was equal to the value being used as the dry weight moisture percentage; set to the moisture value of the treated sample). The results in the second set values were equivalent to the weight of the original sample after drying (only a change in the moisture value). The results in the third set would be the proximate values of the “dry” sample.

Calculations of percent loss of lipid or protein content resulting from mechanical or enzymatic treatments were performed in a similar manner to the conversion of wet weight proximate values to dry weight proximate values above. The dry weight raw proximate values (as calculated above) were converted to the treated (mechanical or enzymatic) proximate values through an intermediate set of values (set 2, as described above). Solver parameters were started with an objective set to the ash value in the third set of values, to be equal to the proximate ash value of the treated sample; the moisture value of the third set was restricted to the proximate moisture value of the treated sample; the ash value of the third set was restricted to values less than or equal to the beginning “dried” sample. The solver function was repeated for lipid and protein, and all three repeated until all four numbers in the third set of values equaled the treated proximate values (the sum of differences, between set 3 and treated values, was <0.01). The percent loss of lipid or protein was then calculated by dividing the difference between the “dried” raw sample proximate value and the optimized second set value, divided by the dried raw sample proximate value, times 100. If the solver function was restricted so the third set of values are kept within 5% of the treated proximate values, it produced an optimized set with fewer repetitions.

### 2.4 Amino acid analysis

Amino acid profiles were determined by the AAA Service Laboratory Inc. (Boring, OR). Samples were hydrolyzed with 6 M HCl and 2% phenol at 110°C for 22 hr. Amino acids were quantified using the Beckman 6300 amino acid analyzer (Beckman Coulter, Brea, CA) with postcolumn ninhydrin derivatization. To minimize, methionine losses oxygen was removed by evacuation of the hydrolysis tubes that contained samples and acid for 10 min prior to putting them in the hydrolysis oven. Cysteine and tryptophan were not determined as separate hydrolysis procedures are required, which increases analysis cost. Two samples of each type of tissue were analyzed for amino acid composition.

### 2.5 Mineral analysis

Elemental analysis was conducted at the University of Missouri‐Columbia College of Agriculture, Food and Natural Resources, Chemical Laboratory on dried samples sent for analysis. Samples were wet digested according to AOAC official method 968.08‐D (b) with nitric acid and perchloric acid. After dilution, the filtered samples were introduced into an ICP‐OES (AOAC 985.01‐(A,B,D)).

### 2.6 Statistical analysis

Enterprise Guide, version 5.1 (SAS, Inc., Cary, NC), was used to conduct the analysis of variance (ANOVA) and Tukey's test on the means data derived from this experimental study. *p* < 0.05 was the relevant significance value used on the data accumulated.

## 3 RESULTS AND DISCUSSION

### 3.1 Effect of process treatments on proximate analysis of Catfish frame bone meal

The percent moisture was highest in the untreated raw frames (13.8%) and was significantly different from the treatments in this study (Table 1). Pressure wash, 30‐min Alcalase treatment, and the 60‐min Alcalase treatment yielded comparable moisture content and showed no significant difference among these respective treatments.

**Table 1:** Proximate composition of Catfish frames with various processing aids

	Raw frame	Pressure wash	Enzyme (30 min)	Enzyme (60 min)
% Moisture	13.78^a^	4.03^b^	4.94^b^	3.11^b^
*SD*	3.34	0.57	0.76	0.86
% Ash	16.87^c^	53.95^b^	ND	62.21^a^
*SD*	0.00	0.04	—	0.00
% Lipids	40.72^a^	8.81^b^	1.58^c^	1.53^c^
*SD*	0.26	2.12	0.24	0.05
% Protein	32.52^a^	34.72^a^	32.70^a^	32.60^a^
*SD*	2.06	0.40	0.32	0.58

Notes

Means on the same row with the same superscripts are not significantly different from each other.

ND: not determined.

Lipid content was also observed to be the largest in the untreated frames (40.7%), and it was significantly different from the treatments. The pressure wash treatment was significantly higher than the enzyme treatments. Alcalase was noted to be quite efficient in removing lipid from the Catfish frames; however, no significant difference was evidenced between the 30‐ and the 60‐min treatments. The reported lipid content of Catfish frame (Bechtel et al., 2017), when converted to similar moisture content, was 42 percent, similar to the 41 percent in this study. Lipid content of tuna frame (prepared similarly to the Catfish raw frame in Table 1) was reported as 11 percent (Abbey, Glover‐Amengor, Atikpo, Atter, & Toppe, 2017) or 27 percent (Istiqlaal, 2017), and for Pollock and Cod, values of 4 and 3 percent were reported (after conversion to 4 percent moisture; Bechtel, 2003)—much lower than the 41 percent in these Catfish samples. In comparison with the treated Catfish frame (1.53 percent lipid), cleaned bones (head and frame) from Cod and Blue whiting had a lipid content of 1.14 and 4.91 percent, respectively, but Salmon and Trout had 38.12 and 34 percent, respectively (Toppe et al., 2007).

Protein content of the frame bone showed no significance between the untreated frame and the three treatments, and no significance between the three treatments. However, it was observed that the pressure wash treatment expressed more percent protein than the other treatments in this study. Protein content of the raw frame (33 percent) was very similar to that reported for Catfish frame (converted to a moisture content of 13.8 percent) with 34 percent (Bechtel et al., 2017). Raw Tuna frame is reported to have 29 percent protein (Abbey et al., 2017) or 9 percent (Istiqlaal, 2017). Protein content of cleaned bone (head and frame) of Cod and Blue whiting was reported as 36 and 42 percent, respectively, and Salmon and Trout had 29 and 31 percent, respectively (Toppe et al., 2007), compared to the 33 percent found for Catfish in this report.

Percent ash for the 30‐min enzyme treatment of Catfish frame bone meal was not determined. However, the 60‐min treatment yielded the highest value of 62.2 percent, significantly higher than the pressure wash treatment. Both treatments were significantly higher than the untreated frame, with 17 percent. Ash content of the raw frame (17 percent) was larger than that reported (Bechtel et al., 2017) in Catfish frame (11 percent). For Tuna frame, ash content was reported to be 44 percent (Abbey et al., 2017). The 62 percent ash for the 60‐min enzyme treated was larger than that reported for Cod, Blue whiting, Salmon, or Trout (53, 45, 26, and 27 percent, respectively; Toppe et al., 2007).

From the results in Table 1, with the decrease in lipids corresponding with no change in protein content and a large increase in ash content, it was calculated that the pressure wash percentages correspond to an approximate loss of 93 percent of original lipid and 67 percent of original protein. This calculates to a 1.4:1 lipid/protein mixture that was removed from the frame bone. For the 60‐min enzyme treatment, the material lost from the frame bone calculates as approximately 99 percent total lipid and 73 percent total protein in a similar 1.4:1 ratio. Therefore, the tissue that was attached to the frame, and removed by the treatments, was preferentially lipids. The resulting bone product was low in lipid content and high in protein and mineral (ash) content, ideal for a nutritional supplement or ingredient, and an especially good choice to counter micronutrient‐deficient diets of those who suffer with obesity. Dietary intakes of calcium and iron have been found to below the recommended dietary allowance for individuals preparing for bariatric surgery (Frame‐Peterson, Megill, Carobrese, & Schweitzer, 2017).

### 3.2 Effect of process treatments on proximate analysis of Catfish head bone meal

Since the 30 and 60 min enzyme treatments of Catfish frames were not significantly different, for the Catfish head treatment, shorter time periods were examined. Moisture content of both the 5‐ and 30‐min enzyme treatments of the Catfish head bone were significantly larger than the 0‐min enzyme treatment (Table 2); however, only the 30‐min treatment was larger than the untreated head bone.

**Table 2:** Proximate composition of Catfish head bone after enzyme hydrolysis

	Raw head^a^	Enzyme (0 min)	Enzyme (5 min)	Enzyme (30 min)
% Moisture	4.01^bc^	3.85^c^	4.24^b^	5.77^a^
*SD*	0.02	0.37	0.34	0.26
% Ash	20.1^d^	36.93^c^	43.02^b^	50.58^a^
*SD*	0.96	1.78	1.44	0.91
% Lipids	29.2^a^	12.47^b^	9.86^c^	6.58^d^
*SD*	2.57	0.54	1.02	0.15
% Protein	47.3^a^	46.11^a^	42.60^b^	38.17^b^
*SD*	1.44	1.19	0.58	0.95

Notes

Means on the same row with the same superscripts are not significantly different from each other.

* Data obtained from Bechtel et al. (2017) with conversion to 4% moisture content.

With 29.2 percent lipid, the untreated head was significantly larger than the three enzyme treatments. The 5‐min enzyme treatment was significantly smaller than the 0‐min treatment, and the 30‐min treatment was significantly smaller than the 5‐min treatment, showing a progressive loss of lipid during enzyme treatment. The lipid content of head bone from Pollock, Cod, and Salmon was reported as 6, 4, and 11 percent, respectively (after conversion to 4% moisture; Bechtel, 2003). These values differ greatly from the Catfish raw head, and more closely match the enzyme treated values seen in Table 2.

Protein content of the untreated head bone and the 0‐min enzyme treatment were not significantly different, but they were both significantly larger than the 5‐min or 30‐min treatments, which were not significantly different. Protein content of Pollock, Cod, and Salmon head bone was reported as 70, 75, and 47 percent, respectively (after conversion to 4% moisture; Bechtel, 2003).

### 3.3 Effect of process treatments on mineral content of Catfish frame bone meal

On average, the Catfish frame mineral content, seen in Table 3, shows the 30‐ and 60‐min enzyme treatments to be significantly larger than the pressure wash or untreated frame bone. However, sodium was an exception, with the pressure wash being significantly larger than either enzyme treatment. Potassium in the untreated frame bone was significantly larger than any of the three treatments. For iron, there was not a significant difference between untreated or treated samples. The 60‐min enzyme treated frame bone contained 25 percent calcium and a 2.2 ratio for Ca:P. Raw Pollock and Cod frame bone was reported to contain 5.9 and 6.0 percent calcium, respectively (Bechtel & Johnson, 2004), similar to the Raw Catfish Frame value of 6.3 percent (Table 3).

**Table 3:** Concentration of minerals from Catfish frames with various processing aids (dry wt basis)

	Raw frame	Pressure wash	Enzyme (30 min)	Enzyme (60 min)
Calcium (%)	6.33^c^	21.27^b^	24.30^a^	24.80^a^
*SD*	0.39	1.36	0.30	0.35
Phosphorus (%)	3.27^c^	9.85^b^	11.27^a^	11.47^a^
*SD*	0.21	0.54	0.12	0.15
Sodium (%)	0.27^ab^	0.30^a^	0.23^b^	0.22^b^
*SD*	0.03	0.02	0.00	0.01
Magnesium (%)	0.13^c^	0.34^b^	0.40^a^	0.42^a^
*SD*	0.01	0.02	0.01	0.01
Potassium (%)	0.51^a^	0.24^b^	0.17^b^	0.17^b^
*SD*	0.06	0.01	0.02	0.02
Copper (ppm)	<0.2	<0.20	<0.20	<0.20
*SD*	—	—	—	—
Zinc (ppm)	48.87^c^	118.67^b^	134.33^a^	138.67^a^
*SD*	3.25	3.21	7.51	2.08
Manganese (ppm)	8.89^b^	24.60^a^	32.17^a^	27.23^a^
*SD*	0.44	7.46	4.24	7.62
Nickel (ppm)	<0.20	<0.20	0.90^a^	0.43
*SD*	—	—	0.71	0.25
Iron (ppm)	6.16^a^	8.68^a^	2.46^a^	5.33^a^
*SD*	2.92	4.35	2.55	1.62

Notes

Means on the same row with the same superscripts are not significantly different from each other.

* *n* = 2 (one rep was undetectable).

### 3.4 Effect of process treatments on mineral content of Catfish head bone meal

The general trend for mineral content of head bone for the three enzyme treatments was a significant increase with increasing treatment time from 0 to 5 to 30 min (Table 4). However, sodium, potassium, and nickel did not show a difference between treatment times, and iron showed a reverse direction, with significant decreases with enzyme treatment time. The 30‐min enzyme treated head bone contained 20 percent calcium and a 2.1 ratio for Ca:P. Raw Pollock and Cod head bone was reported to contain 6.6 and 5.6 percent calcium, respectively (Bechtel & Johnson, 2004), but the 0‐min enzyme treatment in Table 4 is different from raw Catfish head bone since it was heated in water and filtered through a mesh.

**Table 4:** Concentration of minerals from Catfish heads with various processing aids (dry wt basis)

	Enzyme (0 min)	Enzyme (5 min)	Enzyme (30 min)
Calcium (%)	14.79^c^	17.14^b^	19.52^a^
*SD*	0.58	0.38	0.47
Phosphorus (%)	7.05^c^	8.12^b^	9.24^a^
*SD*	0.21	0.12	0.17
Sodium (%)	0.42^a^	0.40^a^	0.42^a^
*SD*	0.00	0.01	0.00
Magnesium (%)	0.26^c^	0.30^b^	0.34^a^
*SD*	0.00	0.01	0.00
Potassium (%)	0.33^a^	0.29^a^	0.25^a^
*SD*	0.00	0.02	0.00
Copper (ppm)	<0.10	<0.10	<0.10
*SD*	—	—	—
Zinc (ppm)	114.00^c^	122.50^b^	135.50^a^
*SD*	1.41	0.71	0.71
Manganese (ppm)	14.30^c^	16.50^b^	18.45^a^
*SD*	0.00	0.28	0.21
Iron (ppm)	76.50^a^	59.05^b^	45.80^c^
*SD*	2.40	0.78	3.96
Nickel (ppm)	0.31^a^	0.65^a^	0.54^a^
*SD*	0.06	0.61	0.12

Note

Means on the same row with the same superscripts are not significantly different from each other.

### 3.5 Effect of process treatments on amino acid content of Catfish frame bone meal

As seen in Table 5, essential amino acids (EAA) showed a significant decrease from untreated frame bone (33.2%) to pressure wash (21.5%) to enzyme treatments (19.4%–19.7%). Hydroxyproline (Hyp) concentration showed a reverse trend with a significant increase from the untreated frame (3.9%) to pressure wash (7.1%) to enzyme treatments (7.7%–8.0%), with both enzyme treatments having no significant difference. Proline also significantly increases from the untreated frame to the three treatments. Almost all other amino acids show a significant decrease from the untreated frame to the three treatments. The most abundant amino acid in the 60‐min enzyme treatment was glycine, alanine, proline, glutamic acid, and hydroxyproline on a mole basis. The high content of hydroxyproline is due in part to the connective tissue in bone. The hydroxyproline content of Cod, Salmon, and Trout bones was reported as 5, 6, and 6 percent (Toppe et al., 2007), compared to 8 percent for the enzyme treatments and 7 percent for the pressure wash in this study. The relative amino acid content of the treated Catfish frame bone is very similar to collagen, and therefore if hydrolyzed, may be useful as a dietary supplement or functional food or beverage for the benefit of joint and bone health or enhancement of skin health.

**Table 5:** Amino acids of Catfish frames with various processing aids (wt/wt%)

	Raw frame	Pressure wash	Enzyme (30 min)	Enzyme (60 min)
ALA (A)	7.53^b^	9.04^a^	9.27^a^	9.25^a^
*SD*	0.34	0.21	0.05	0.04
ARG (R)	7.10^b^	7.70^a^	7.73^a^	7.79^a^
*SD*	0.13	0.13	0.05	0.02
ASP (D)	9.07^a^	6.71^b^	6.23^b^	6.32^b^
*SD*	0.46	0.27	0.03	0.11
GLU (E)	12.82^a^	9.99^b^	9.76^b^	9.76^b^
*SD*	0.36	0.44	0.04	0.05
GLY (G)	12.30^b^	20.10^a^	21.16^a^	21.04^a^
*SD*	1.54	0.97	0.08	0.15
HIS (H)*	1.80^a^	1.24^b^	1.15^b^	1.16^b^
*SD*	0.16	0.07	0.01	0.02
HYP (Z)	3.92^c^	7.09^b^	8.03^a^	7.72^ab^
*SD*	0.75	0.27	0.13	0.23
ILE (I)*	3.77^a^	2.17^b^	1.87^b^	1.86^b^
*SD*	0.39	0.19	0.01	0.04
LEU (L)*	6.01^a^	3.49^b^	3.03^b^	3.04^b^
*SD*	0.55	0.3	0.01	0.05
LYS (K)*	7.20^a^	4.09^b^	3.83^b^	3.98^b^
*SD*	0.65	0.28	0.11	0.07
MET (M)*	2.43^a^	1.63^b^	1.55^b^	1.54^b^
*SD*	0.18	0.09	0.01	0.02
PHE (F)*	3.46^a^	2.41^b^	2.16^b^	2.22^b^
*SD*	0.2	0.12	0.16	0.02
PRO (P)	6.68^b^	11.38^a^	11.99^a^	11.95^a^
*SD*	0.79	0.54	0.08	0.12
SER (S)	4.39^a^	4.19^b^	4.22^b^	4.19^b^
*SD*	0.01	0.11	0.02	0.02
THR (T)*	4.20^a^	3.13^b^	2.92^b^	2.95^b^
*SD*	0.28	0.14	0.01	0.04
TYR (Y)	2.68^a^	1.58^b^	1.48^b^	1.50^b^
*SD*	0.19	0.14	0.07	0.04
VAL (V)*	4.30^a^	3.33^b^	2.90^b^	2.97^b^
*SD*	0.32	0.07	0.1	0.09
EAA*	33.17^a^	21.49^b^	19.41^c^	19.72^c^
*SD*	1.08	0.51	0.22	0.14
NEAA	66.49^c^	77.78^b^	79.87^a^	79.52^a^
*SD*	2.02	1.29	0.21	0.33

Notes

Means on the same row with the same superscripts are not significantly different from each other.

EAA, essential amino acids; NEAA, nonessential amino acids.

*Essential amino acids.

### 3.6 Effect of process treatments on amino acid content of Catfish head bone meal

Table 6 shows the EAA significantly decreases from 0‐min enzyme treatment (30.3%) to 5 min (28.3%) to 30 min (25.1%) in Catfish head bone meal. Hydroxyproline and proline show a reversed trend with increasing concentrations with longer enzyme treatment, from 4.4 percent to 6.1 percent.

**Table 6:** Amino acids of Catfish heads with various processing aids (wt/wt%)

	Enzyme (0 min)	Enzyme (5 min)	Enzyme (30 min)
ALA (A)	7.61^c^	7.82^b^	8.45^a^
*SD*	0.07	0	0.03
ARG (R)	7.12^b^	7.25^b^	7.54^a^
*SD*	0.06	0.08	0
ASP (D)	8.33^a^	7.97^b^	7.29^c^
*SD*	0.1	0.06	0.02
GLU (E)	12.50^a^	12.09^b^	10.45^c^
*SD*	0.01	0.14	0
GLY (G)	13.89^c^	15.09^b^	17.64^a^
*SD*	0.32	0.24	0.02
HIS (H)*	1.93^a^	1.85^b^	1.67^c^
*SD*	0.02	0.01	0
HYP (Z)	4.39^c^	4.99^b^	6.09^a^
*SD*	0.15	0.13	0
ILE (I)*	3.11^a^	2.86^b^	2.47^c^
*SD*	0.07	0.02	0.01
LEU (L)*	5.69^a^	5.17^b^	4.47^c^
*SD*	0.1	0.13	0.01
LYS (K)*	5.86^a^	5.43^b^	4.75^c^
*SD*	0.07	0.16	0.01
MET (M)*	2.18^a^	2.09^a^	1.89^b^
*SD*	0.04	0.02	0.01
PHE (F)*	3.38^a^	3.17^b^	2.83^c^
*SD*	0.09	0.02	0
PRO (P)	7.44^c^	8.25^b^	9.80^a^
*SD*	0.09	0.08	0.06
SER (S)	4.61^a^	4.57^a^	4.27^b^
*SD*	0.01	0.05	0.03
THR (T)*	4.09^a^	3.88^b^	3.41^c^
*SD*	0.07	0.06	0.01
TYR (Y)	2.87^a^	2.66^b^	2.37^c^
*SD*	0.07	0.07	0.01
VAL (V)*	4.08^a^	3.88^b^	3.57^c^
*SD*	0.07	0.01	0
EAA*	30.32^a^	28.33^b^	25.06^c^
*SD*	0.20	0.022	0.02
NEAA	68.67^c^	70.51^b^	73.81^a^
*SD*	0.40	0.34	0.08

Notes

Means on the same row with the same superscripts are not significantly different from each other.

EAA: essential amino acids; NEAA: nonessential amino acids.

*Essential amino acids.

## 4 CONCLUSION

Bone from Catfish frames was prepared using two methods: (a) use of a proteolytic enzyme to digest the nonbone tissues and (b) after boiling the frames, removal of the nonbone tissues with high‐pressure water. Both methods produced clean bone products with some differences in ash (62% and 54%) and lipid content (2% and 9%); however, percent protein was similar at 33% and 35% for the two methods, respectively. The amino acid profiles were similar for both methods with high levels of hydroxyproline present, and elemental composition was also similar. Because the enzymatic treatment had significantly larger percent of ash (containing a larger percent of calcium, phosphorus, and zinc) and significantly lower percentage of lipid, the product from this method would be preferred. Bone from Catfish heads was prepared by digestion of the nonbone tissues with a proteolytic enzyme and collection of the bone with a sieve. After the longest digestion period, the dried head bone was 51% mineral, 38% protein, and 7% lipid. The amino acid profile had high levels of hydroxyproline and lower levels of many essential amino acids, consistent with connective tissue proteins. With increase enzymatic hydrolysis time, the percent calcium and phosphorus increased indicating a greater removal of nonbone tissue. Results from this study will be used in the development of new value‐added food and feed ingredients from Catfish bone.

## CONFLICT OF INTEREST

The authors declare that they do not have any conflict of interest. This article is a US Government work and is in the public domain in the USA. Mention of a trademark or proprietary product is for identification only and does not imply a guarantee or warranty of the product by the US Department of Agriculture. The US Department of Agriculture prohibits discrimination in all its programs and activities on the basis of race, color, national origin, gender, religion, age, disability, political beliefs, sexual orientation, and marital or family status.

## AUTHOR CONTRIBUTION

Peter J. Bechtel (Food Technologist)—conception and design, interpretation of data, drafting of manuscript; Michael A. Watson (Food Technologist)—acquisition of data, drafting of manuscript; Jeanne M. Lea (Food Technologist)—analysis of data; Karen L. Bett‐Garber (Food Technologist)—analysis of data; John M. Bland (Chemist)—analysis of data, drafting of manuscript.

## ETHICAL STATEMENT

This study does not involve any human or animal testing.
